# 11β-Hydroxysteroid dehydrogenase type 1 amplifies inflammation in LPS-induced THP-1 cells

**DOI:** 10.22038/IJBMS.2023.67927.14852

**Published:** 2023-03

**Authors:** Lingli Luo, Dongmei Zhu, Zheng Zhang, Hanjie Zeng, Min Huang, Suming Zhou

**Affiliations:** 1 Department of Geriatrics Intensive Care Unit, The First Affiliated Hospital of Nanjing Medical University. NO.300 Guangzhou Road, Nanjing, 210029, Jiangsu Province, China

**Keywords:** 11β-Hydroxysteroid- dehydrogenase type 1, BVT.2733, Glucocorticoid, Inflammation, THP-1 cells

## Abstract

**Objective(s):**

The role of glucocorticoids as anti-inflammatory and immune-stimulatory drugs has been widely reported. However, the role of 11β-hydroxysteroid dehydrogenase type 1 (11β-HSD1), which catalyzes the conversion of inactive cortisone into active cortisol, in inflammation remains unclear. This study aimed to examine the mechanism of actions of 11β-HSD1 in lipopolysaccharide (LPS)-induced THP-1 cells.

**Materials and Methods:**

The gene expression of 11β-HSD1 and pro-inflammatory cytokines was detected via RT-PCR. The protein expression of IL-1β in cell supernatants was detected via ELISA. Oxidative stress and mitochondrial membrane potential were assessed using a reactive oxygen species (ROS) kit and a mitochondrial membrane potential (MMP) kit, respectively. The expression of Nuclear Factor- Kappa B (NF-κB) and mitogen-activated protein kinase (MAPK) was detected via western blotting.

**Results:**

Elevated levels of 11β-HSD1 contributed to the expression of inflammatory cytokines, whereas BVT.2733, a selective 11β-HSD1 inhibitor, ameliorated inflammatory responses, ROS, and mitochondrial damage in LPS-stimulated THP-1 cells. Furthermore, cortisone and cortisol, which are the substrate and product of 11β-HSD1, respectively, showed biphasic responses and induced the expression of pro-inflammatory cytokines at a low concentration in both LPS-stimulated or untreated THP-1 cells. The enhanced inflammation was attenuated by co-treatment with BVT.2733 and the glucocorticoid receptor (GR) antagonist RU486, but not in those treated with the mineralocorticoid receptor (MR) antagonist spironolactone. Overall, the results indicate that 11β-HSD1 amplifies inflammatory responses by activating the NF-κB and MAPK signaling pathways.

**Conclusion:**

Inhibition of 11β-HSD1 may serve as a potential therapeutic target against the excessive activation of inflammation.

## Introduction

Inflammation is a coping strategy of the immune system to protect the body from injury and infection. However, the dysregulated release of pro-inflammatory cytokines can lead to a ‘cytokine storm’. Owing to prolonged and uncontrolled accumulation, inflammatory cytokines can spill over into the circulation and result in multi-organ dysfunction (1), eventually leading to death caused by inflammation-associated cytokine storms (2). 

As a class of steroid hormones, glucocorticoids are widely used for immunoregulation and anti-inflammatory treatment. Glucocorticoids can be classified as natural and synthetic hormones, with the former including cortisone and cortisol, and the latter including dexamethasone, prednisone, and methylprednisolone (3). Much of meta-analyses and studies have demonstrated the immunosuppressive properties of glucocorticoids. However, studies have also shown that glucocorticoids aggravate inflammation by enhancing the enzymatic activity of 11β-HSD1 (4-6). Therefore, the effects of glucocorticoids on inflammation remain controversial in clinical practice. 

11β-HSD1 regulates the intracellular levels of glucocorticoids mainly by converting intrinsically inactive glucocorticoids (cortisone in humans and 11-dehydrocorticosterone in mice) into an active form (cortisol in humans and corticosterone in mice), thereby catalyzing the regeneration of active glucocorticoids to enhance cellular activity (7). It is important for glucocorticoids to maintain the balance between activity and inactivity through expression of 11β-HSD1 at different sites (8-10). Previous studies have shown that hormones, metabolism, and inflammatory cytokines regulate the expression of 11β-HSD1 (11, 12). IL-1β and TNF-α up-regulate 11β-HSD1 expression in various cells, and the cell activation state dynamically regulates the enzymatic activity of 11β-HSD1 (13). Selective inhibition or deficiency of 11β-HSD1 can improve various metabolic disorders and insulin sensitivity, and prevent preterm birth (6, 10, 14-18). However, distinct from previous works, the exact role and mechanism of action of 11β-HSD1 in inflammation have been poorly understood. 

In the present study, we examined the role of 11β-HSD1 in inflammation using BVT.2733, a selective 11β-HSD1 inhibitor, for the treatment of LPS-induced inflammation. The results demonstrated the protective effects of BVT.2733 against cell injury and the mechanism of action of 11β-HSD1 during inflammation *in vitro*.

## Materials and Methods

***Cell culture and experimental reagents***

THP-1 cells (American Type Culture Collection, USA) were incubated in the RPMI 1640 medium (Gibco, USA) supplemented with 10% heat-inactivated fetal bovine serum (Gibco, USA) and 1% streptomycin and penicillin (Gibco, USA) under standard conditions (95% humidified air of 5% CO_2_ at 37 ^°^C). LPS (Sigma, USA) at a concentration of 100 μg/ml was dissolved in sterile water. Cortisone, cortisol, RU486, and spironolactone (MCE, China) at a concentration of 1 mM were respectively dissolved in PBS. BVT.2733 (Selleck, USA) at a concentration of 100 mM was dissolved in DMSO.

***RNA extraction and RT-PCR assay***

Total mRNA was extracted from THP-1 cells using the TRIzol reagent (Ambion, USA). Briefly, 1 μg of total RNA was reverse transcribed into cDNA using HiScript III RT SuperMix (Vazyme, China) according to the manufacturer’s instructions. Real-time PCR was measured using ChamQ SYBR Green qPCR Master Mix (Vazyme, China) on the Step One Plus^TM^ Real-time PCR System (Applied Biosystems, USA) according to the manufacturer’s instructions. RNA levels were estimated using the 2^-ΔΔCT^ method and normalized to GAPDH (19). The sequences of primers used for PCR are listed in (Table 1).

***Western blotting***

THP-1 cells were seeded into 6-well plates at a density of 2×10^5^ cells/ml. Total protein was extracted from cells lysed with ice-cold RIPA buffer (Beyotime, China) and quantified using the BCA Protein Assay Kit (Thermo Fisher Scientific, USA). The isolated protein (20 μg) was loaded onto 12% SDS-PAGE gels for electrophoresis and transferred onto PVDF membranes (Millipore, USA). The membranes were blocked with 5% non-fat dried milk and incubated with primary antibodies against p-JNK (Proteintech, China, 1:2000), JNK (Proteintech, China, 1:4000), p-P38 (Proteintech, China, 1:2000), P38 (Proteintech, China, 1:4000); GAPDH, ERK, p-ERK, NF-ΚB, and p-NF-ΚB (Cell Signaling Technology, USA, 1:1000) overnight at 4 ^°^C. The following day, the membranes were incubated with appropriate secondary antibodies (rabbit anti-mouse (1:10000, Proteintech, China) or goat anti-rabbit (1:10000, Proteintech, China) antibodies) for 2 hr at 4 ^°^C. thereafter, protein bands were visualized using an ECL detection system (Tanon, China). 

***Enzyme-linked immunosorbent assay***

The concentration of IL-1β in THP-1 cell supernatants was estimated using an enzyme-linked immunosorbent assay (ELISA) kit (Proteintech, China) according to the manufacturer’s instructions.

***Measurement of oxidative stress***

THP-1 cells were seeded into 12-well plates at a density of 5×10^5^ cells/ml and incubated for 6 hr at 37 ^°^C. The cells were incubated with or without BVT.2733 one hour before LPS treatment. The cells in the LPS and LPS+BVT.2733 groups were treated with LPS for 6 hr at 37 ^°^C. Intracellular ROS levels were detected using the Reactive Oxygen Species Assay Kit (Beyotime, China) in accordance with the manufacturer’s instructions. Briefly, the cells were incubated with the fluorescent probe DCFH-DA (10 μM) for 20 min at 37 ^°^C in the dark and washed with cold PBS. The fluorescence intensity was imaged using a fluorescence microscope (OLYMPUS, Japan). Five fields were randomly examined by two researchers blindly.

***Assessment of mitochondrial membrane potential***

The mitochondrial membrane potential (MMP) of THP-1 cells was measured using the Mitochondrial Membrane Potential and Apoptosis Detection Kit (Beyotime, China) in accordance with the manufacturer’s instructions. Apoptosis and cell viability were detected using the Hoechst 33342 stain and green fluorescent probe Annexin V-FITC, respectively. Meanwhile, MMP was detected using Mito-Tracker Red CMXRos. The cells were centrifuged for 5 min at 800 g and resuspended in PBS. A total of 1×10^4^ cells obtained via centrifugation were resuspended in 188 μl Annexin V-FITC binding buffer, 5 μl Annexin V-FITC, and 2 μl Mito-Tracker Red CMXRos. The mixed solution was incubated for 20 min, and the fluorescence intensity of cells was imaged using a fluorescence microscope (OLYMPUS, Japan). 

***Statistical analysis***

Experimental data were presented as mean±standard deviation. One-way analysis of variance (ANOVA) followed by Tukey’s test was used for comparing the data of more than two groups. Statistical analysis was performed using the GraphPad Prism software (version 9.0). A *P*-value of <0.05 indicated significant differences.

## Results

***Inhibition of 11β-HSD1 by BVT.2733 ameliorates inflammation in LPS-induced THP-1 cells***

**Figure 1:**
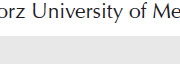
Inhibition of 11β-HSD1 by BVT.2733 ameliorates inflammation in LPS-induced THP-1 cells

As shown in (Figures 1A, B, and C), THP-1 cells were treated with LPS at different concentrations, the mRNA expression of 11β-HSD1, IL-1β and IL-6 increased in a concentration-dependent manner, and the highest mRNA expression of 11β-HSD1 was measured in cells treated with 1×10^3^ ng/ml of LPS. Therefore, the LPS concentration of 1×10^3^ ng/ml was used for subsequent experiments to examine the mechanisms underlying 11β-HSD1-mediated inflammatory responses. It is noteworthy that the quantitative analysis of mRNA expression depended on the state of THP-1 cells and the batch of LPS.

THP-1 cells treated with LPS showed markedly increased mRNA expression of IL-1β, IL-6, IL-8, IL-10, COX-2, and TNF-α. However, after the cells were treated with BVT.2733, which selectively inhibits the enzymatic activity of 11β-HSD1, the mRNA expression of these pro-inflammatory cytokines, except TNF-α, was decreased (Figure 1D). Additionally, BVT.2733 treatment alleviated the protein expression of IL-1β in cell supernatants (Figure 1E). These results indicate that RNA expression is consistent with protein secretion to a certain extent. 

***BVT.2733 effectively attenuates LPS-induced ROS and mitochondrial membrane potential***

**Figure 2:**
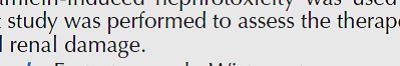
BVT.2733 effectively attenuates LPS-induced ROS and mitochondrial membrane potential

LPS-induced inflammation leads to oxidative stress and decreases MMP (20). The fluorescence intensity of ROS in LPS-induced THP-1 cells increased almost two-fold compared with the control group (Figures 2A and B). However, co-treatment with LPA and BVT.2733 attenuated ROS production in THP-1 cells. Increased ROS levels can interfere with normal mitochondrial function by decreasing MMP (21). Consistently, the number of densely stained apoptotic cells and the green fluorescence intensity increased after LPS treatment (Figures 2C-E), indicating that LPS reduces cell viability and induces apoptosis. The lower intensity of red fluorescence indicated the reduced stability of MMP in LPS-induced THP-1 cells compared with control cells. However, these negative effects were abolished by BVT.2733.

***Low-dose endogenous glucocorticoids amplified inflammatory responses in LPS-induced THP-1 cells***

**Figure 3:**
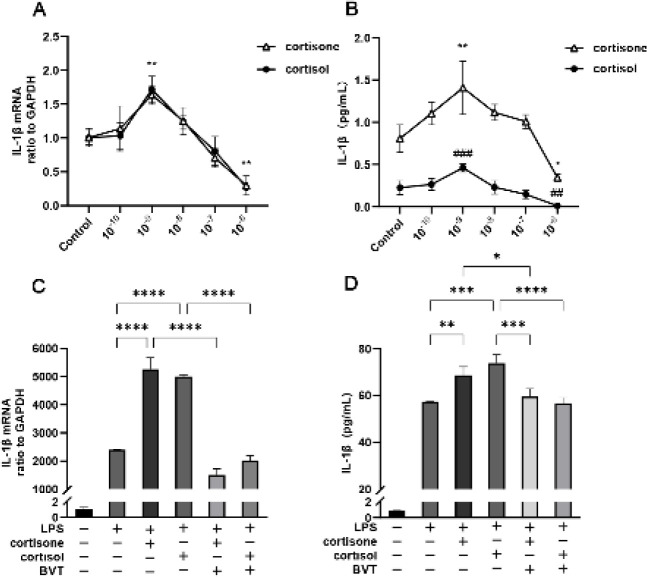
Low-dose endogenous glucocorticoids amplified inflammatory responses in LPS-induced THP-1 cells

11β-HSD1 catalyzes the regeneration of cortisol from biologically inactive cortisone in immune cells. THP-1 cells were treated with cortisone or cortisol at various concentrations. Both cortisone and cortisol showed a biphasic response with peak stimulatory effects on IL-1β at a concentration of 1 nM and suppression at a concentration of 1000 nM compared with the control group (Figure 3A). The biphasic impact of cortisone and cortisol on protein levels in cell supernatants is shown in (Figure 3B). The absolute IL-1β concentrations of the two control groups were different possibly due to different algebraic THP-1 cells.

Furthermore, THP-1 cells in the activated state were examined. Pre-treatment with 1 nM cortisone for 24 hr (low physiological glucocorticoid concentration) further amplified IL-1β expression in LPS-stimulated THP-1 cells (Figure 3C). Similar results were obtained when cortisol was used as the substrate. However, treatment with BVT.2733 attenuated the increased expression of IL-1β. These results were consistent with the protein expression of IL-1β examined via ELISA (Figure 3D). 

***11β-HSD1 amplifies inflammation through GR***

**Figure 4:**
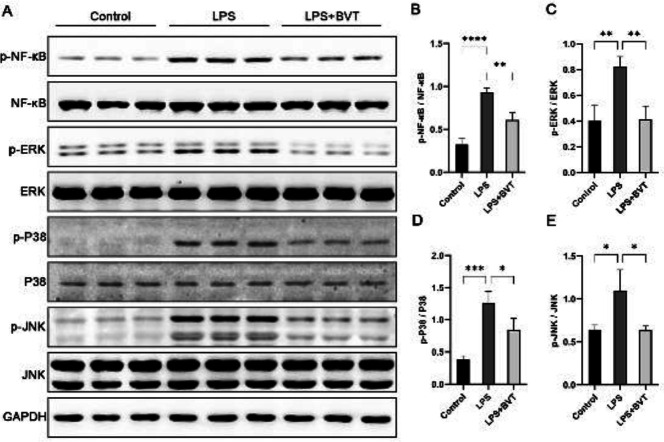
11β-HSD1 amplifies inflammation through glucocorticoid receptor (GR)

As an amplifier of glucocorticoid activity, 11β-HSD1 converts the inactive cortisone into active cortisol. In THP-1 cells, the physiological activity of endogenous glucocorticoids is mediated not only by GR but also MR (22). Therefore, we directly treated the low-concentration glucocorticoids induced THP-1 cells with either GR antagonist RU486 or MR antagonist spironolactone. Cortisone and cortisol generated biphasic responses, and the stimulatory effects of low-concentration glucocorticoids were ameliorated in cells pre-treated with RU486, with or without LPS (Figures 4A, B, E, and F). However, spironolactone did not affect the expression of IL-1β (Figures 4C, D, G, and H). Therefore, low-dose endogenous glucocorticoids regulated by 11β-HSD1 may amplify inflammatory responses through GR.

***BVT.2733 ameliorates inflammatory responses by inhibiting the NF-*** ***κB*** ***and MAPK pathways***

**Figure 5:**
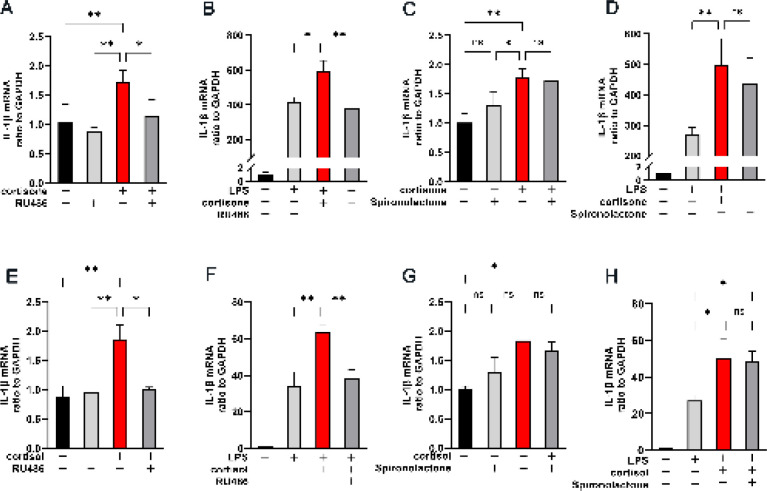
BVT.2733 ameliorates inflammatory responses by inhibiting the NF-κB and MAPK pathways

To elucidate the effects of 11β-HSD1 on inflammatory responses, we examined LPS-induced activation of NF-κB and MAPK, which play a crucial role in oxidative stress and inflammation (23, 24). LPS stimulation activated the phosphorylation of NF-κB and MAPK (ERK, P38, and JNK); however, these effects were reversed after treatment with BVT.2733 (Figure 5).

**Table 1:** Sequences of the primer pairs for qRT-PCR

**Genes**	**Primer sequences（5´→3´）**
IL-1β	F: GTGGCAATGAGGATGACTTGTTC
	R: TAGTGGTGGTCGGAGATTCGTA
IL-6	F: TTCGGCAAATGTAGCATG
	R: AATAGTGTCCTAACGCTCATAC
IL-8	F: CTGATTTCTGCAGCTCTGTG
	R: GGGTGGAAAGGTTTGGAGTATG
IL-10	F: GTGATGCCCCAAGCTGAGA
	R: CACGGCCTTGCTCTTGTTTT
TNF-α	F: CGAGTGACAAGCCTGTAGC
	R: GGTGTGGGTGAGGAGCACACAT
COX-2	F: CAGCACTTCACGCATCAGTT
	R: CGCAGTTTACGCTGTCTAGC
11β-HSD1	F: TGGCTTATCATCTGGCGAAGA
	R: AGGCAGTGGGATACCACCT
GAPDH	F: ACAACTTTGGTATCGTGGAAGG
	R: GCCATCACGCCACAGTTTC

F: forward; R: reverse

(A-C) THP-1 cells were treated with LPS at different concentrations (ng/ml) for 24 hr. The mRNA expression of 11β-HSD1, IL-6, and IL-1β was detected via RT-PCR (*P<0.05, **P<0.01, ***P<0.001, and ****P<0.0001 compared with the control group). (D) THP-1 cells were initially treated with 100 μM BVT.2733 for 1 hr and subsequently treated with 1 μg/ml LPS for 6 hr. The mRNA expression of IL-1β, IL-6, IL-8, IL-10, TNF-α, and COX-2 was detected via RT-PCR. (E) The protein expression of IL-1β was measured via ELISA. Data were analyzed by one-way ANOVA followed by Tukey’s multiple comparisons test (n=3). (ns, non-significant; *P<0.05, ** *P<*0.01, ***P<0.001, and ****P<0.0001). BVT, BVT.2733

(A) ROS production was analyzed by detecting the fluorescence of DCFH-DA (n=3). (B) Histograms demonstrating the relative quantitative expression. (C) Apoptotic cells were assessed using Hoechst 33342 (blue) and Annexin V-FITC (green) stains after pre-treatment of cells with BVT.2733 for 1 hr and subsequent treatment with LPS (×20 magnification; scale bar, 100 μm). The MMP of THP-1 cells was examined using Mito-Tracker Red CMXRos (red), and fluorescence intensity was quantified (D and E). (n=3). (*P<0.05, **P<0.01, and ****P<0.0001). BVT, BVT.2733

(A and B) THP-1 cells were treated with cortisone or cortisol at different concentrations, and the mRNA and protein expression of IL-1β were detected via RT-PCR and ELISA, respectively (*P<0.05, **P<0.01 compared with the control group; ^#^*P<*0.01, ^###^*P<*0.001 compared with the control group). (C and D) THP-1 cells were pre-treated with 1 nM cortisone or cortisol for 24 hr with or without 100 μM BVT.2733 and subsequently treated with LPS for 6 hr (n=4). (*P<0.05, **P<0.01, ***P<0.001, and ****P<0.0001). BVT, BVT.2733

(A-D) THP-1 cells were treated with 1 nM cortisone in the presence or absence of RU486 and spironolactone for 24 hr, with or without LPS treatment. The mRNA expression of IL-1β was quantified via RT-PCR. (E-H) THP-1 cells were pre-treated with 1 nM cortisol in the presence or absence of RU486 and spironolactone for 24 hr, with or without LPS treatment. The mRNA expression of IL-1β was quantified via RT-PCR (n=4). (ns, non-significant. *P<0.05, **P<0.01, ***P<0.001, and ****P<0.0001). BVT, BVT.2733

(A) Phosphorylated levels of NF-κB and MAPK were determined in LPS-induced THP-1 cells with or without BVT.2733. (B-E) Quantification of western blotting results represented in (A) (n=4). (*P<0.05, **P<0.01, ***P<0.001, and ****P<0.0001). BVT, BVT.2733

JNK: c-Jun N-terminal kinase; ERK: extracellular regulated protein kinases

## Discussion

Although the pathophysiological role of 11β-HSD1 in metabolic syndrome has been investigated (14, 19), its potential role in mediating the function of human immune cells and endogenous glucocorticoids during inflammation is still poorly understood. Monocytes are crucial components of the immune system and can synthesize and secrete various cytokines. The THP-1 cell line has been widely used to investigate the inflammatory responses of monocytes (25, 26). Thieringer *et al.* (27) reported that the expression of 11β-HSD1 is up-regulated after LPS-, IL-3- or IL-4-induced differentiation of monocytes into macrophages. What’s more, the increased expression of 11β-HSD1 may enhance the level of glucocorticoids and curb inflammation. With this foundation, we studied the role and mechanism of 11β-HSD1 in LPS-induced THP-1 cells. The results indicated that 11β-HSD1 promoted inflammatory responses, ROS, and mitochondrial damage by activating the NF-κB and MAPK signaling pathways. 

In a recent study, 11β-HSD-knockout mice were found to recruit more inflammatory cells and aggravate inflammation by locally reduced levels of cortisol, resulting in a retarded resolution of the immune response in arthritis induced by K/BxN serum (28); while the level of plasma cortisol is normal though 11β-HSD1 is up-regulated during chronic inflammation (including rheumatoid arthritis) (11). Pharmacological inhibition of 11β-HSD1 can attenuate the levels of pro-inflammatory mediators and increase the survival rate in endotoxemia (4). Our data showed that 11β-HSD1 was expressed in a concentration-dependent manner in LPS-induced THP-1 cells, and inhibition of 11β-HSD1 by BVT.2733 ameliorated the pro-inflammatory mediators. Thus, BVT.2733 exerts therapeutic effects against inflammation by suppressing the production of pro-inflammatory cytokines. 

Mitochondria are the principal organelles involved in cell survival, energy production, and death regulation. ROS triggers complex cellular signaling pathways, including the production of cytokines and aggravation of mitochondrial dysfunction during inflammatory responses (21). Consistently, in this study, ROS production and apoptosis were increased, and MMP was markedly decreased in LPS-induced THP-1 cells. However, these effects were reversed after BVT.2733 treatment. Increased expression of the intracellular glucocorticoid-reactivating enzyme 11β-HSD1 contributes to the dysfunction of THP-1 cells. Inhibition of 11β-HSD1 may protect against LPS-induced ROS, apoptosis, and MMP; reduce mitochondrial damage; and protect cells from injury. 

Previous studies have demonstrated that inflammatory cytokines are induced by low-concentration glucocorticoids in 3T3-L1 adipocytes, BV-2 cells, and human epidermal keratinocytes (5, 29, 30). Increased mRNA and protein expression levels of IL-6 have been reported in BV-2 cells treated with 25 nM 11-dehydrocorticosterone or 250 nM corticosterone (30). However, these changes reflect the local pro-inflammatory responses produced by murine microglial cell lines, and the mechanisms underlying the 11β-HSD1-mediated increase in inflammation remain unclear. Our data showed that inflammatory cytokines were increased in THP-1 cells with 1 nM cortisone or cortisol. The concentration of pro-inflammatory cytokines in cells stimulated with cortisone or cortisol differs from that in our study, possibly owing to the differences in 11β-HSD1 metabolism in different cells. Park *et al*. (4) investigated that inhibition of 11β-HSD1 activity can up-regulate the expression of HO-1 and provide protection against LPS-induced cell injury, spleen injury, and animal death via inhibition of inflammation. Our data showed that 11β-HSD1, which is a key enzyme of glucocorticoid regeneration, not only promoted inflammation with a low concentration of cortisone or cortisol in the resting state of the cells but also further amplified inflammatory responses through GR in LPS-induced THP-1 cells. Endogenous glucocorticoids exert biphasic responses, which is an important clue to understanding glucocorticoid-mediated immunoregulation. Inflammatory responses were amplified after treatment with low-concentration cortisone in the presence or absence of LPS and abolished by co-treatment with BVT.2733. Unexpectedly, the inflammatory response enhanced via treatment with low-concentration cortisol was attenuated by BVT.2733 as well. A possible explanation for this phenomenon is as follows: 11β-HSD1 expression can be increased by glucocorticoids (7), and the feed-forward metabolism of 11β-HSD1 mediated by cortisol further increases the abundance of cortisol in THP-1 cells.

The different affinities of the GR and MR for glucocorticoids are due to different tissues and glucocorticoids concentration (22). We showed that 11β-HSD1 amplifies pro-inflammatory responses in THP-1 cells treated with low-concentration endogenous glucocorticoids in the presence of RU486, but not in the presence of the spironolactone, suggesting that 11β-HSD1 amplifies inflammation at least partially through GR, which is consistent with previous studies (18, 31). 

Pro-inflammatory cytokines, oxidative stress, apoptosis, and mitochondrial damage intensify the phosphorylation of the NF-κB and MAPK pathways. These functional proteins relay, amplify, and integrate signals that may cause severe tissue damage and eventually lead to organ failure (32). In this study, LPS elevated the phosphorylated levels of NF-κB and MAPK, whereas BVT.2733 reversed these effects. It suggested that BVT.2733 alleviated the inflammation of LPS-induced THP-1 cells maybe by suppressing NF-κB and MAPK pathways.

## Conclusion

11β-HSD1 amplifies inflammatory responses, ROS production, and mitochondrial damage in LPS-induced THP-1 cells by activating the NF-κB and MAPK signaling pathways. Inhibition of 11β-HSD1 may represent a potential therapeutic strategy for preventing the excessive activation of inflammation.

## Authors’ Contributions

SMZ and MH designed the experiments; LLL performed the laboratory experiments, collected data, and wrote this article; DMZ, ZZ, and HJZ discussed the results and strategy; LLL, MH, and SMZ supervised, directed, and managed the study; LLL, DMZ, ZZ, HJZ, MH, and SMZ approved the final version to be published.

## Conflicts of Interest

The authors have no conflicts of interest to declare. 
